# Therapeutic frontiers in viral myocarditis: targeting inflammation, viruses, oxidative stress, and myocardial repair

**DOI:** 10.3389/fimmu.2025.1643502

**Published:** 2025-08-14

**Authors:** Jingyao Xu, Xuanjia Chen, Xia Guan, Haiying Zhang, Yan Liu, Min Zhang

**Affiliations:** ^1^ Engineering Research Center of Tropical Medicine Innovation and Transformation of Ministry of Education, School of Pharmacy, Hainan Medical University, Haikou, China; ^2^ Hainan Provincial Key Laboratory of Research and Development on Tropical Herbs, Hainan Medical University, Haikou, China

**Keywords:** viral myocarditis, inflammation intervention, gene therapy, antioxidant, myocardial repair

## Abstract

Viral myocarditis (VMC) is a life-threatening inflammatory cardiomyopathy with a global incidence rate of 10–22 per 100,000 people. It is the most common clinical manifestation of myocardial inflammation. Myocardial cell injury and fibrosis are the pathological characteristics of VMC. Coxsackievirus B3 (CVB3), parvovirus B19 (PVB19), Severe Acute Respiratory Syndrome coronavirus 2 (SARS-CoV-2), and adenovirus (AdV) are the main causes that induce viral myocarditis. Among them, CVB3 has become the main pathogen, accounting for more than 50% of the confirmed cases of VMC. The clinical manifestations of this disease are extensive, ranging from asymptomatic carriers to sudden cardiac death caused by acute decompensated heart failure and arrhythmia. Current therapeutic strategies for VMC focus on four key approaches: (1) Anti-inflammatory interventions targeting inflammatory cells and mediators; (2) Antiviral therapies employing gene editing, viral protease inhibitors, and RNA polymerase inhibitors; (3) Myocardial protection through tissue repair promotion and nutritional support; (4) Oxidative stress mitigation using antioxidants. This article will systematically summarize the progress of VMC management in recent years and provide personal insights for VMC management.

## 1 Introduction

Myocarditis accounts for approximately 20% of adolescent mortality (1) and is classified as the third most prevalent etiology of cardiovascular death in young athletes (6% incidence), surpassed only by coronary artery anomalies (17%) and hypertrophic cardiomyopathy (36%) (2, 3). The disease manifests with marked clinical heterogeneity (4), ranging from mild presentations (chest pain, palpitations, low-grade fever, exertional dyspnea) to fulminant cardiogenic shock (hypotension, peripheral hypoperfusion) or lethal arrhythmias (sustained ventricular tachycardia/fibrillation), with reported mortality rates of 10%-20% (5). A subset of patients reports prodromal upper respiratory or gastrointestinal infections 1–3 weeks preceding symptom onset (6), while those with localized inflammatory foci may remain clinically silent. Pathogenetically, myocarditis arises from diverse infectious (viral, bacterial, rickettsial) and non-infectious triggers (pharmacotoxic agents, giant cell myocarditis, sarcoidosis), with CVB3 infection representing the predominant etiology across all demographic groups. By diagnostic criteria, myocarditis constitutes a non-ischemic inflammatory cardiomyopathy characterized by lymphocytic infiltration and myocardial necrosis on histopathology (7).

Given this significant disease burden and etiological complexity, establishing a definitive viral myocarditis diagnosis becomes clinically imperative yet methodologically challenging. The pronounced clinical heterogeneity-spanning subclinical presentations to fulminant cardiogenic shock-necessitates correlating epidemiological patterns with precision diagnostic frameworks. Consequently, contemporary guidelines mandate systematic integration of histopathological evidence and multimodal assessment to address diagnostic ambiguities inherent in VMC’s variable manifestations, particularly given CVB3’s predominance across demographic strata.

**Figure 1:**
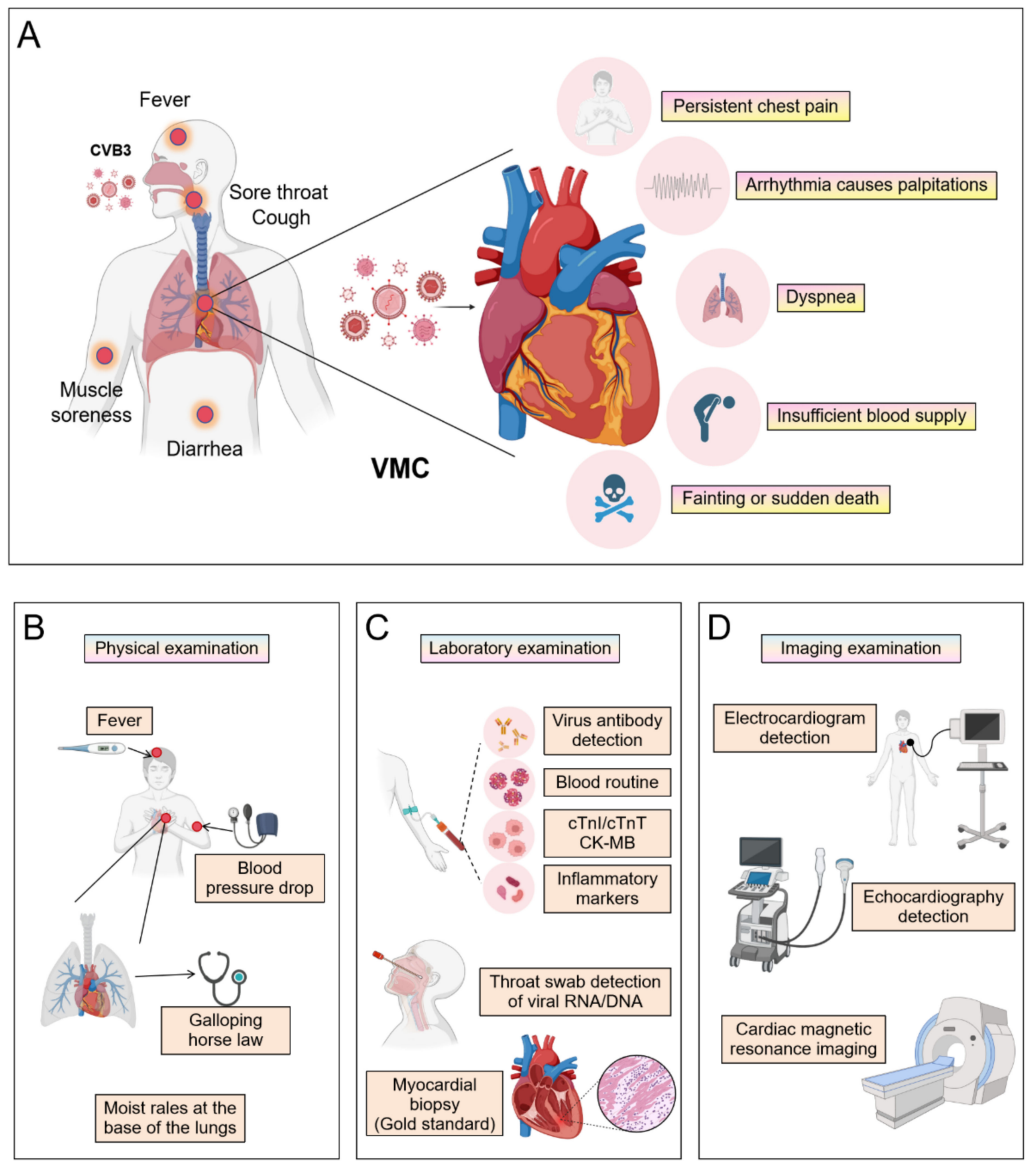
The clinical symptoms and diagnosis of VMC. (By Biorender). **(A)** The manifestations that occur when a virus invades the human body and the symptoms that may appear after invading the heart. The patient underwent **(B)** a physical examination, **(C)** laboratory examinations, and **(D)** imaging examinations.

VMC clinical diagnosis requires a comprehensive multimodal approach (**Figure 1**). This process begins with evaluating characteristic symptoms- such as chest pain, arrhythmias, or heart failure manifestations-alongside elevated serum biomarkers, including cardiac troponin I/T (cTnI/cTnT) and N-terminal pro-B-type natriuretic peptide (NT-proBNP). Subsequent non-invasive imaging assessments involve cardiac magnetic resonance (CMR) with T1/T2 mapping and late gadolinium enhancement (LGE) sequences to characterize myocardial inflammation, edema, and necrosis, while echocardiography quantifies functional impairment through left ventricular ejection fraction (LVEF) depression and wall motion abnormalities. Ischemic etiology must then be excluded via coronary angiography, supplemented by endomyocardial biopsy (EMB) demonstrating lymphocytic infiltrates with viral genome detection via polymerase chain reaction (PCR) or *in situ* hybridization in refractory cases. Definitive diagnosis ultimately adheres to modified Lake Louise Criteria and WHF/ESC consensus guidelines, integrating histopathological, immunological, and functional evidence while rigorously excluding alternative cardiomyopathies.

VMC is pathologically defined by focal or diffuse myocardial inflammation (8), marked by significant infiltration of inflammatory cells-predominantly myeloid cells and T lymphocytes-into cardiac tissue (9). Sustained myocardial inflammation drives structural remodeling through fibrotic deposition and hypertrophic adaptations, progressively replacing functional myocardium with collagenous tissue. This pathological cascade ultimately impairs systolic or diastolic function and precipitates arrhythmogenesis. Furthermore, chronic systemic inflammation secondary to myocarditis accelerates atherosclerotic progression, elevating predisposition to lethal ischemic complications (10).

Current therapeutic paradigms prioritize four core strategies: (1) implementation of targeted antiviral interventions, (2) prescription of structured physical rest to reduce myocardial stress, (3) application of standardized heart failure management protocols, and (4) modulation of cytokine-mediated inflammatory responses within cardiac tissue. Notably, modulating the pathological inflammatory cascade remains a critical therapeutic priority requiring advanced intervention strategies.

Despite notable advances in VMC management, current therapeutic paradigms remain predominantly confined to anti-inflammatory interventions with suboptimal efficacy. Given myocardial inflammation’s centrality in VMC pathology, conventional strategies persistently target inflammatory cascades through two complementary approaches: (1) modulating specific immune cell populations to enhance anti-inflammatory precision, and (2) sustaining immunomodulation to remodel the inflammatory microenvironment. While this represents a strategic shift from direct antiviral action toward host immune regulation, such anti-inflammatory-centric frameworks exhibit inherent limitations. Consequently, achieving improved clinical outcomes necessitates innovative targeting beyond inflammation-particularly through emerging gene therapies, mesenchymal stem cell applications, and redox-modulating strategies. This review critically synthesizes recent advances in VMC classification and therapeutics, with focused analysis on CVB3-induced myocarditis pathogenesis and intervention gaps.

## 2 Classification of VMC

**Figure 2:**
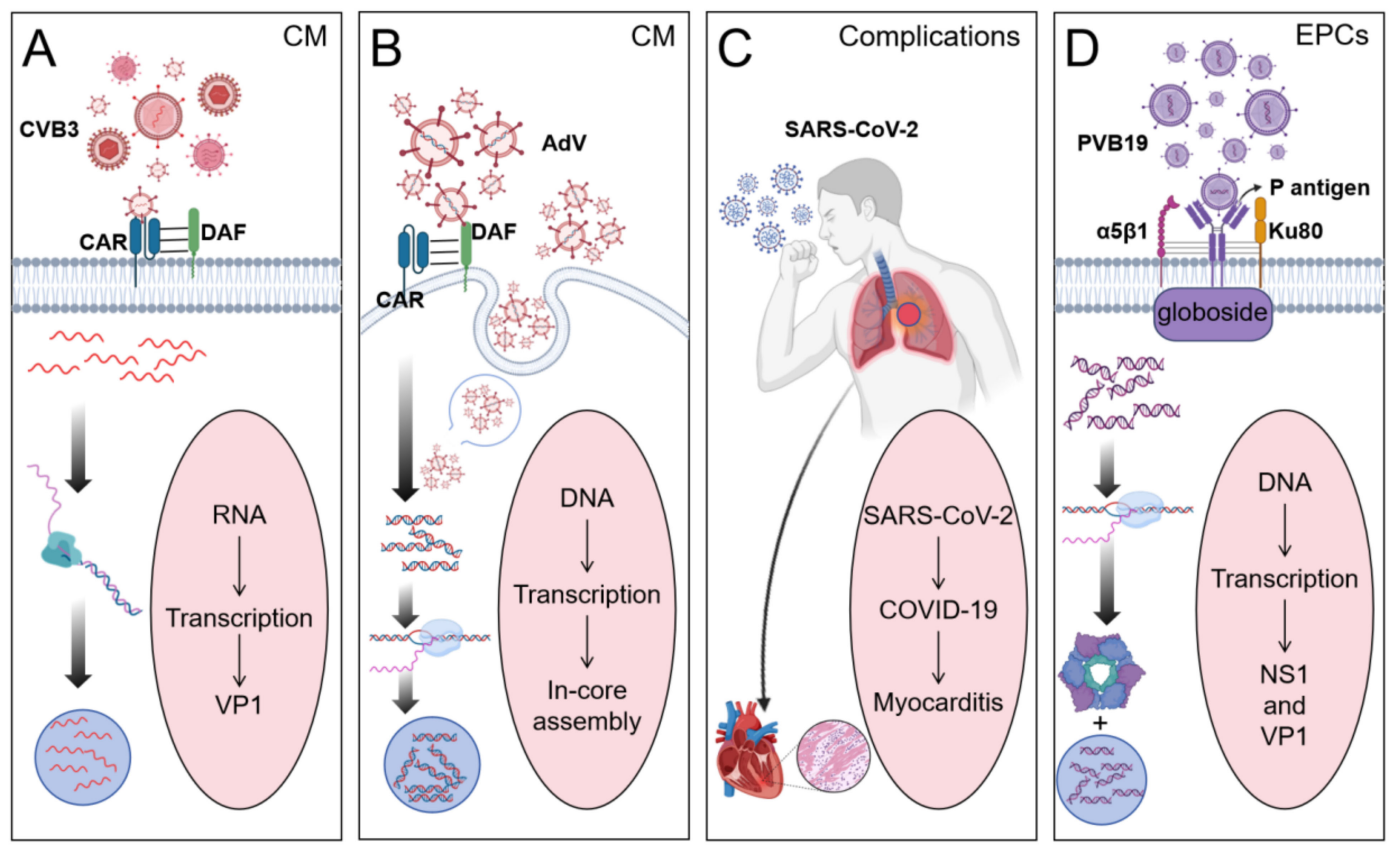
Four different viruses cause the occurrence of myocarditis. (By Biorender). **(A)** CVB3 binds to the Coxsackievirus-adenovirus receptor (CAR) in cardiomyocytes (CM) to promote viral internalization and binds to the Decay acceleration factor (DAF) on the cell surface to initiate viral adhesion. Viruses replicate within host cells to form replication complexes. **(B)** Adenovirus promotes viral internalization by binding to CAR on the surface of CM through the head domain of its fiber protein, and can also enter CM through endocytosis. Viral DNA replicates within cells and assembles in the nucleus. **(C)** Human infection with SARS-CoV-2 induces COVID-19, leading to myocarditis as a complication. **(D)** PVB19 promotes viral adhesion to the cell membrane by recognizing and binding to the P antigen (globoside) on the surface of endothelial progenitor cells. The α5β1 integrin, while the Ku80 protein acts as a coreceptor, enhancing the binding efficiency of the virus to host cells. The non-structural protein NS1 has helicase activity and can promote the replication of viral DNA.

VMC is mediated by diverse etiological agents, among which four viral pathogens have been identified as predominant contributors: CVB3, PVB19, SARS-CoV-2, and AdV. This review focuses on these four viral and delineates their distinct mechanisms of host invasion (**Figure 2**).

### 2.1 Coxsackievirus B3

The predominant etiological agent of VMC is recognized as CV (11), with enterovirus CVB3 identified as the most prevalent subtype during the 1980s-1990s. Viral entry into cardiomyocytes occurs through binding to the constitutively expressed transmembrane receptor coxsackie-adenovirus receptor (CAR) (12), facilitated by the decay-accelerating factor (DAF or CD55) co-receptor. This interaction initiates direct myocardial injury and cytoskeletal disruption (13), subsequently provoking persistent immune dysregulation post-viral clearance. Notably, the CVB3/28 strain demonstrates exclusive CAR-dependent infectivity (14), while the CVB3 Variant PD exhibits replication capability in DAF/CAR-deficient cells, with potential involvement of alternative adhesion molecules such as heparan sulfate proteoglycans (15). Current research delineates three principal mechanisms underlying CVB3-induced tissue pathology: direct viral cytotoxicity (16), infection-triggered inflammatory cascades, and their synergistic amplification of myocardial damage (17). The resolution mechanisms for CVB3-induced cardiac inflammatory cascades remain a critical unresolved question in pathogenesis research.

### 2.2 Parvovirus B19

PVB19 has been established as an additional causative agent of myocarditis alongside CVB3 (18). While typically manifesting as a mild, self-limiting condition, PVB19 infection demonstrates significant potential for triggering severe systemic sepsis and hematological complications. The virus can provoke acute cardiac infection during high-titer viremia (19) and establish persistent myocardial reservoirs with reactivation potential (20). Classified as a vasotropic pathogen, PVB19 primarily targets myocardial vascular endothelial cells and erythroid progenitor cells without directly infiltrating cardiomyocytes (21). Notably, endothelial cell infection may initiate secondary cardiomyocyte apoptosis through indirect mechanisms (22). Following acute symptom resolution, the virus transitions to a latent phase. The clinical implications of persistent PVB19 myocardial reservoirs remain controversial, requiring further investigation to clarify their prognostic significance (23).

### 2.3 Severe Acute Respiratory Syndrome Coronavirus 2

The Coronaviridae family members Middle East Respiratory Syndrome Coronavirus (MERS-CoV), Severe Acute Respiratory Syndrome Coronavirus (SARS-CoV), and SARS-CoV-2 all demonstrate cardiac involvement with documented myocarditis development (24). The pathogenic mechanism involves three key elements: viral tropism for angiotensin-converting enzyme 2 (ACE2) receptors, cytokine-mediated myocardial injury, and autoimmune responses targeting cardiac antigens (25). SARS-CoV-2-induced myocarditis represents a severe cardiovascular complication of COVID-19, presenting with heterogeneous clinical manifestations ranging from chest discomfort and exertional dyspnea to cardiac arrhythmias and syncopal episodes (26). Notably, this complication exhibits pan-demographic susceptibility and may demonstrate prolonged latency, potentially extending therapeutic timelines. These characteristics underscore the critical need for optimized early detection protocols and targeted intervention strategies requiring urgent optimization through comprehensive clinical research.

### 2.4 Adenovirus

AdV represents a cardiotropic pathogen (27), classified as a non-enveloped dsDNA virus with approximately 50 characterized serotypes. These variants demonstrate clinical heterogeneity ranging from self-limiting respiratory infections to fatal systemic manifestations (28). Notably, human AdV serotypes 2 and 5 are predominantly associated with acute myocarditis and inflammatory cardiomyopathy development (29, 30). Viral entry into cardiomyocytes occurs through CAR binding (12), inducing myocardial injury and cytoskeletal disruption. This structural degradation initiates persistent immune activation persisting post-viral clearance (13). The sustained cardiac immune activation presents a significant therapeutic challenge, necessitating further investigation into targeted resolution mechanisms.

## 3 The treatment of VMC induced by CVB3

CVB3 has emerged as the primary etiological agent of VMC. Effective clinical management hinges on early intervention and targeted mitigation of inflammatory cascades. Recent advancements in preclinical model research and clinical evidence accumulation have facilitated significant therapeutic progress, enabling systematic development and evaluation of multiple innovative strategies. Current experimental therapies encompass several targeted approaches: inflammatory cell modulation therapy, inflammatory mediator regulation therapy, genetic intervention strategies, stem cell-based regenerative therapies, and redox homeostasis restoration protocols.

### 3.1 Inflammatory cell intervention

Inflammatory cell modulation therapy represents a therapeutic strategy that targets inflammation-associated pathologies through precise regulation of inflammatory cell dynamics, including population control, functional modification, and activity modulation. The therapeutic paradigm focuses on three critical cellular processes: targeted modulation of inflammatory cell infiltration patterns, activation states, and polarization phenotypes (including macrophages, T lymphocytes, and neutrophils). This approach achieves therapeutic effects through three primary mechanisms: suppression of pathological inflammation, alleviation of clinical manifestations, and facilitation of regenerative tissue remodeling.

**Figure 3:**
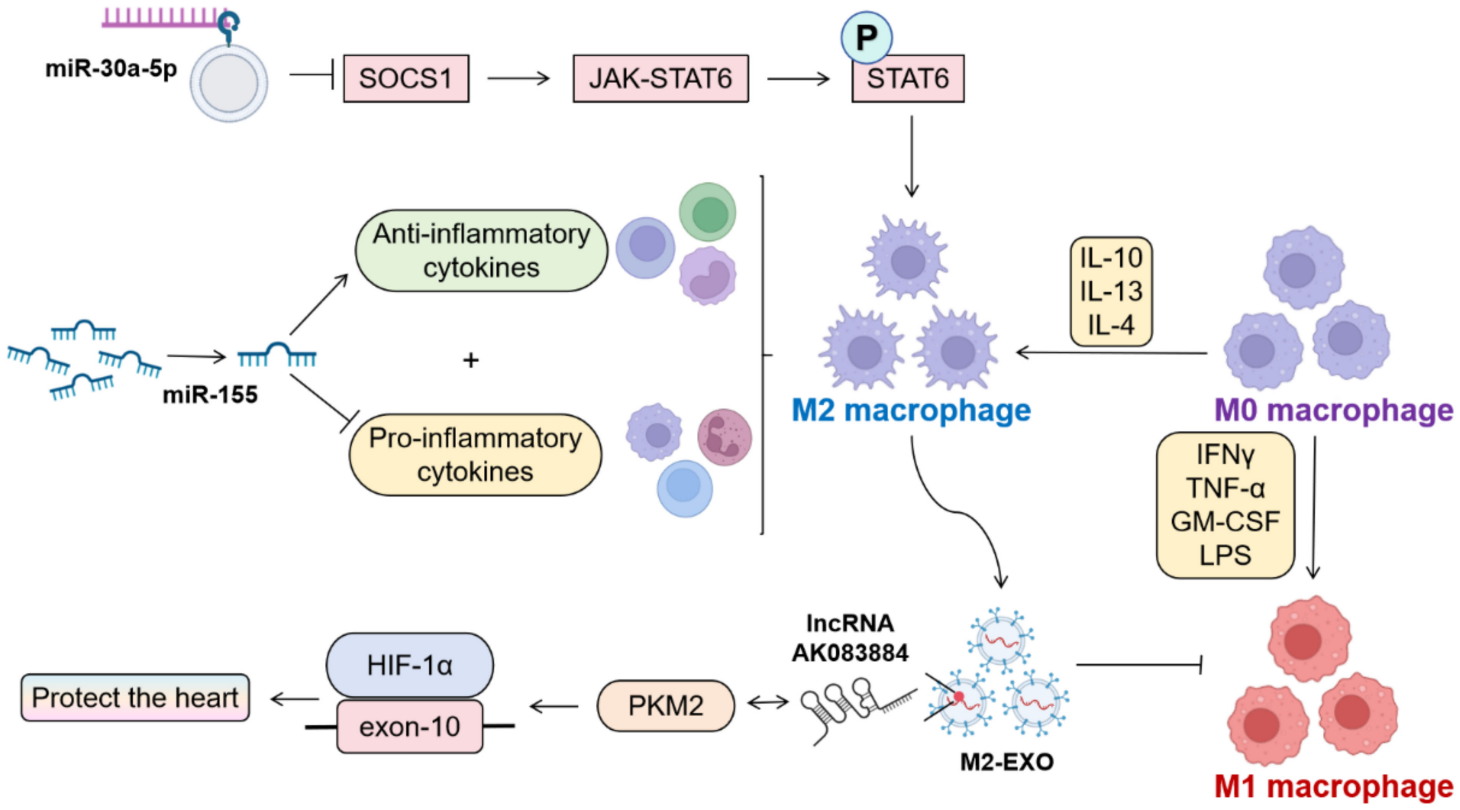
Influencing factors of M1/M2 polarization in macrophages. (By Biorender). Therapeutic suppression of miR-30a-5p and miR-155 has been shown to facilitate M2 macrophage polarization, thereby mitigating viral myocarditis-induced cardiac injury. Exosomes derived from M2-polarized macrophages (M2-Exo) demonstrate cardioprotective effects through dual mechanisms: modulation of the pyruvate kinase M2-hypoxia-inducible factor 1-alpha (PKM2-HIF-1α) complex activity and concomitant downregulation of M1 phenotypic surface markers and pro-inflammatory cytokine expression.

Macrophage M1/M2 polarization equilibrium critically governs tissue homeostasis, with polarization dynamics being intrinsically linked to cellular metabolic profiles (**Figure 3**). M1 macrophages undergo profound metabolic reprogramming (31), while M2 counterparts predominantly utilize oxidative phosphorylation (OXPHOS) pathways (32). Experimental evidence demonstrates that both microrNA-155 (miR-155) silencing and miR-30a-5p silencing exert convergent therapeutic effects through enhanced M2 polarization. Notably, miR-30a-5p operates via SOCS1-mediated mechanisms to attenuate viral myocarditis (33–35). miR-155^−/−^ mice exhibit elevated anti-inflammatory cytokines with concomitant pro-inflammatory cytokine suppression post-CVB3 challenge. M2-derived exosomes (M2-EXO) demonstrate cardioprotective efficacy in CVB3-induced VMC through lncRNA AK083884/PKM2 axis-mediated metabolic reprogramming, modulating HIF-1α transcriptional activity via PKM2-HIF-1α complex formation (36, 37).These findings collectively establish macrophage polarization modulation as a pivotal therapeutic paradigm in myocarditis management, with targeted polarization strategies representing promising clinical interventions.

Beyond macrophage polarization-mediated anti-inflammatory effects, T-cell-mediated immunity demonstrates a synergistic therapeutic potential. The activation of macrophages initiates the inflammatory cascade. Infiltrating pro-inflammatory macrophages demonstrate synergistic interaction with CD8^+^ effector T lymphocytes (38), with T-cells directly exacerbating myocardial injury via IFN-γ secretion, which induces cardiomyocyte apoptosis and disease progression through Spleen focus-forming virus (SFFV) proviral integration oncogene 1 (SPI1) transcription factor upregulation (39).

Study shows thrombospondin-2 (TSP-2) exerts critical cardioprotective effects in CVB3-induced myocarditis through immunomodulation of regulatory T lymphocytes (Tregs) (40). TSP-2 deficiency markedly elevates murine mortality, exacerbates myocardial inflammation, tissue necrosis, and collagen deposition, induces substantial infiltration of CD3^+^, CD4^+^ and CD8^+^ T lymphocytes within cardiac tissue. Meanwhile, TSP-2 overexpression activates Treg populations, attenuates lymphocyte infiltration, reduces necrotic lesions, and enhances cardiac functional recovery via interacting with CD47 (41). These findings elucidate the TSP-2/Treg axis as a central immunoregulatory mechanism, revealing therapeutic potential for both TSP-2 gene augmentation or Treg amplification strategies to mitigate myocardial injury and improve clinical outcomes through restoration of immunoregulatory homeostasis (42).

Neutrophil activation and extracellular trap (NET) formation exhibit marked elevation during VMC acute phase pathogenesis (43). Experimental evidence demonstrates that lymphocyte antigen 6 complex locus G (anti-Ly6G) antibody-mediated neutrophil depletion attenuates myocardial necrosis and leukocyte infiltration while suppressing monocyte recruitment and pro-inflammatory macrophage differentiation. Furthermore, genetic ablation of peptidylarginine deiminase 4-dependent (PAD4-dependent) NET generation substantially mitigates cardiac injury and reduces myocardial myeloid cell infiltration, particularly monocyte and macrophage populations. These collective findings establish neutrophil activity suppression and NET response inhibition as viable therapeutic approaches to ameliorate disease pathology and myocardial inflammation (44).

Macrophages, T lymphocytes, and neutrophils collectively exert critical immunomodulatory functions in VMC pathogenesis. These immunocytes coordinate through synergistic interactions to maintain immune homeostasis, attenuate myocardial inflammation, and preserve cardiac functional integrity. Targeting immunocyte modulation therapies require comprehensive investigation to optimize their therapeutic potential and establish standardized protocols for clinical VMC management.

### 3.2 Inflammatory mediator modulation therapy

**Figure 4:**
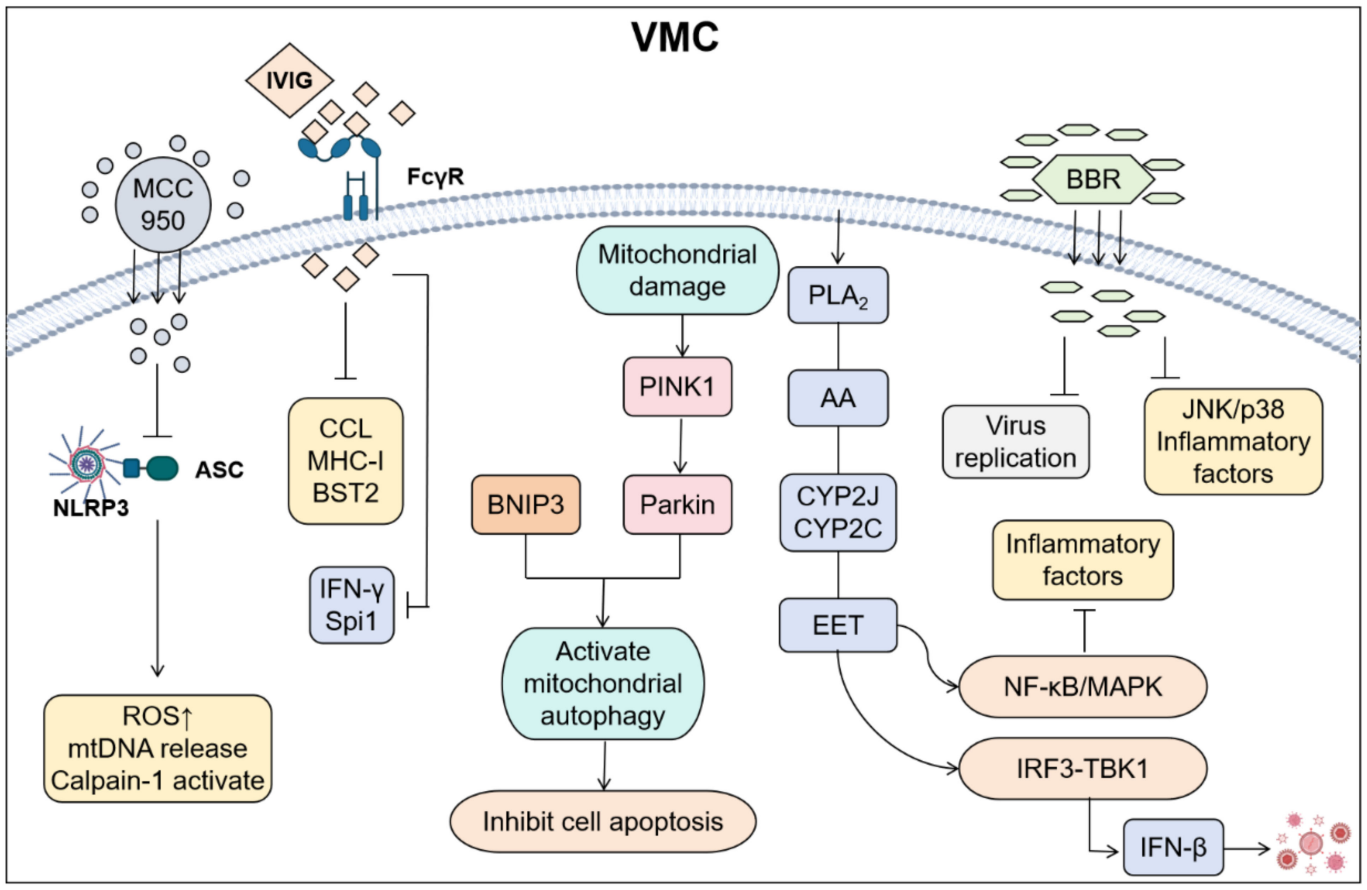
Cardiac injury was reduced by regulating inflammatory mediators in VMC. (By Biorender). MCC950 alleviates damage by inhibiting the binding of NLRP3 to Apoptosis-associated speck-like protein containing a CARD (ASC); The IgG of IVIG binds to FcγR on the surface of immune cells such as macrophages and neutrophils through the Fc segment, inhibiting excessive inflammatory responses and reducing the release of inflammatory mediators; Parkin/BNIP3 alleviates apoptosis by activating mitochondrial autophagy; EET inhibits the activation of NF-κB, reduces the expression of inflammatory cytokines, and promotes the interaction between GSK3β and TBK1. Coordinate type I interferon signal transduction to improve cardiac function and alleviate inflammatory responses; BBR can inhibit viral replication and suppress the JNK/p38 pathway and inflammatory factors.

Modulating inflammatory mediators is a therapeutic strategy that targets key signaling molecules involved in the inflammatory response. This approach aims to optimize the inflammatory response, minimize tissue damage, and facilitate tissue repair and functional recovery by regulating components such as cytokines, metabolites, and the extracellular matrix (**Figure 4**). This therapeutic strategy demonstrates significant potential for application across various inflammatory diseases, including cardiovascular diseases, autoimmune diseases, neurodegenerative disorders, and tumor-associated inflammation. The administration of drugs such as immunoglobulins and glucocorticoids can effectively suppress excessive immune responses, thereby reducing myocardial cell damage (45).

Inflammasome inhibition diminishes inflammatory mediator production and release, thereby modulating inflammatory cascades. Current research confirms that the NLRP3 inflammasome exhibits marked upregulation in cardiomyocytes and infiltrating cardiac macrophages during VMC (46). This molecular complex acts as a cytoplasmic sensor that recognizes both exogenous pathogens and endogenous damage-associated molecular patterns. Subsequently, it initiates Caspase-1-dependent proteolytic cascades, which drive the maturation of interleukin-1β (IL-1β) and IL-18. These cascades further orchestrate pyroptotic cell death pathways (47). CVB3 structural proteins VP1 and VP2 serve as crucial NLRP3 activation triggers. Pharmacological inhibition using MCC950 significantly mitigates macrophage/T-cell infiltration (48), attenuates inflammation, and preserves cardiac function. Furthermore, either calpain enzymatic inhibition or upregulation of muscle-specific membrane repair protein MG53 effectively suppresses NLRP3 inflammasome activation (49, 50), ameliorating inflammatory responses and pyroptosis. Conversely, MG53 knockdown exacerbates both inflammation and pyroptotic cell death (51). Emerging evidence suggests that modulating mitochondrial quality control mechanisms and its crosstalk with inflammatory signaling pathways offers novel therapeutic avenues for myocarditis management.

Parkin, an E3 ubiquitin ligase, plays a pivotal role in mitophagy regulation. In CVB3-induced VMC murine models, Parkin/BNIP3-mediated mitophagy is initiated but autophagic flux remains impaired, with disrupted autophagosome-lysosome fusion (52–54). Parkin silencing increases mortality and worsens cardiac dysfunction in CVB3-infected mice. This impairment disrupts mitophagic clearance, leading to accumulated damaged mitochondria and enhanced apoptosis (55). Additionally, Parkin deficiency dysregulates both mitophagy and inflammatory responses in neonatal rat cardiomyocytes. Mechanistically, Parkin preserves cardiac function and modulates inflammation during acute VMC through coordinated regulation of mitophagy and NF-κB signaling. Notably, Parkin deficiency may compromise viral defense and autoimmune myocarditis protection via interleukin-1 receptor accessory protein (IL1RAP) pathway. IL1RAP inhibition using mCAN10 monoclonal antibody substantially attenuates myocardial pathology severity, suppresses inflammatory infiltration, and downregulates IL-1 signaling/chemokine expression (56). Beyond IL1RAP targeting, exogenous anti-inflammatory agents such as epoxyeicosatrienoic acids (EETs) broaden therapeutic options by concurrently modulating NF-κB and type I interferon pathways in myocarditis management.

EETs, bioactive metabolites derived from arachidonic acid through cytochrome P450 (CYP) epoxygenases (57), demonstrate potent anti-inflammatory activity (58, 59). EETs undergo rapid conversion to relatively inactive dihydroxyeicosatrienoic acids (DHETs) via soluble epoxide hydrolase (sEH). sEH inhibitors exhibit therapeutic efficacy through endogenous EETs stabilization. EETs suppress inflammatory cytokine expression by inhibiting NF-κB nuclear translocation and preserve cardiac function in VMC through type I interferon pathway modulation (60–62). Specifically, EETs facilitate molecular interplay between glycogen synthase kinase-3β (GSK3β) and TANK-binding kinase 1 (TBK1), potentiating interferon-β biosynthesis to coordinate type I interferon signaling for enhanced cardiac functional recovery and inflammation resolution (63). Beyond synthetic EET analogs, natural pharmacological agents like berberine offer multi-target therapeutic approaches for myocarditis through concurrent modulation of viral replication mechanisms and inflammatory signaling cascades.

Berberine (BBR), a bioactive isoquinoline alkaloid isolated from *Coptis chinensis*, exhibits multifaceted anti-inflammatory properties. Experimental evidence demonstrates that BBR’s potent inhibition of CVB3 proliferation in HeLa cells while mitigating virus-induced cytopathic effects. Mechanistically, BBR suppresses c-Jun N-terminal kinase (JNK) and p38 mitogen-activated protein kinase (p38 MAPK) activation through reduced phosphorylation, consequently attenuating viral VP1 protein synthesis and double-stranded RNA production (64, 65). In murine CVB3 infection models, BBR administration significantly enhances survival rates, ameliorates myocardial pathology, preserves cardiac function, and reduces viral titers while suppressing pro-inflammatory cytokine release and macrophage infiltration. These findings establish BBR as a novel therapeutic framework for CVB3-induced myocarditis through dual mechanisms of viral replication inhibition and inflammatory response modulation. Unlike dual-targeting phytochemicals that modulate both viral and inflammatory pathways, biopharmaceutical agents (e.g., immunoglobulins) employ polypharmacology approaches. These interventions orchestrate immune cell polarization and facilitate crosstalk coordination between antiviral and inflammatory signaling networks. Consequently, they establish integrated therapeutic paradigms by modulating synergistic immune-inflammatory axis interactions.

Immunoglobulin treatment demonstrates enhanced survival rates in fulminant myocarditis mice (100% vs. 40%), improves cardiac functional parameters, attenuates myocardial inflammation, and balances cytokine profiles by suppressing pro-inflammatory mediators (IL-1β, IL-6, TNF-α) while promoting anti-inflammatory IL-10 expression (66). Single-cell RNA sequencing reveals immunoglobulin’s dual regulatory effects on cardiac immunity: restoring immune cell homeostasis through suppression of excessive innate immune activation, decreasing monocyte and neutrophil infiltration, and enhancing macrophage antigen presentation capacity to optimize antiviral responses. Mechanistically, immunoglobulins coordinate inflammatory regulation through three key pathways: modulating chemotaxis via CCL signaling, optimizing antigen presentation through MHC-I regulation, and fine-tuning antiviral responses via Bone marrow stromal cell antigen 2 (BST2) signaling (67). These findings substantiate immunoglobulin’s therapeutic potential for myocarditis via comprehensive immunomodulation. They demonstrate concurrent improvements in cardiac function and clinical outcomes, while also proposing novel mechanistic insights. These insights provide a foundation for clinical translation.

Beyond immunoglobulins, IVIG demonstrates therapeutic efficacy in VMC through triple mechanisms: viral neutralization, immunomodulation, and anti-inflammatory action. IVIG delivers broad-spectrum antiviral antibodies that directly neutralize viral particles to prevent cardiomyocyte injury. It concurrently suppresses pathological inflammation by downregulating pro-inflammatory mediator release and normalizing dysregulated immune pathways (68, 69). IVIG reduces mortality, suppresses viral load, and restores immune homeostasis via IFN-γ/Spi1. Unlike IVIG’s multi-targeted immune-antiviral synergy, adrenal corticosteroids (e.g., prednisone) primarily exert single-target anti-inflammatory effects through glucocorticoid receptor activation.

Adrenal corticosteroids, including prednisone and methylprednisolone, serve as cornerstone therapies for VMC owing to their potent anti-inflammatory effects. These agents effectively modulate hyperactive immune responses by suppressing inflammatory cell infiltration and limiting the production of pro-inflammatory mediators. However, clinical application requires cautious risk-benefit evaluation due to their dual-edged nature: immunosuppression may compromise host defense mechanisms while inadvertently exacerbating viral proliferation. Modern therapeutic approaches targeting inflammatory mediators have transitioned from isolated anti-inflammatory interventions to integrated strategies addressing virus-immune-metabolic crosstalk. Future advancements require precision-targeted interventions that transition therapeutic goals from symptom management to reversal of pathological remodeling. This shift demands innovations in spatiotemporal target identification and Artificial intelligence-driven drug development. Concurrently, immunophenotype-guided clinical stratification will enable achievable translation of these therapies.

### 3.3 Gene therapy

GT introduces a novel therapeutic paradigm for VMC through modulating of immune response and inflammatory injury. Concurrently, targeted molecular interventions that disrupt viral replication cycles establish precise antiviral therapeutic avenues.

As a core enzymatic component of enteroviral replication, RNA helicase represents a strategic therapeutic target, with its pharmacological inhibition of which demonstrates potent blockade of CVB3 pathogenicity. Enterovirus-2C inhibitor (E2CI) targeting the enteroviral 2C protein through specific binding to effectively suppress ATPase activity and disrupt viral genomic RNA replication (70–72). Mechanistically, E2CI exhibits robust antiviral efficacy *in vitro*, achieving significant suppression of CVB3 proliferation in both HeLa cells (EC50 = 0.32 μM) and primary human cardiomyocytes while maintaining favorable host cell compatibility (CC50 > 50 μM) (73). Translational studies in CVB3-infected murine models confirm therapeutic superiority, with E2CI-treated cohorts demonstrating significantly enhanced survival rates (92% vs. 71%, p < 0.05) accompanied by multimodal cardioprotection: accelerated viral clearance, attenuated myocardial pathology, and preserved cardiac functional parameters. These findings underscore the therapeutic value of viral replication pathway interruption while highlighting complementary intervention opportunities through coordinated modulation of host inflammatory cascades in viral myocarditis management.

Long non-coding RNAs (lncRNAs) constitute a distinct class of non-protein-coding transcripts exceeding 200 nucleotides in length. Experimental evidence demonstrates that long non-coding guanylate-binding protein 9 (lncGBP9) silencing suppresses NF-κB pathway activation, leading to reduced secretion of key pro-inflammatory cytokines (TNF-α, IL-6, IL-1β). *In vitro* validation reveals that lncGBP9 depletion in HL-1 cardiomyocytes significantly enhances cellular viability (p < 0.01) with concomitant decreases in apoptotic indices (p < 0.05), while effectively attenuating both cytokine storm and NF-κB nuclear translocation. These results mechanistically position lncGBP9 as a regulator of inflammatory-apoptotic crosstalk through NF-κB pathway inhibition, suggesting its therapeutic potential for VMC intervention (74). Building upon lncRNA-mediated inflammatory modulation mechanisms, subsequent investigations delineated miRNA’s dual regulatory capacity in balancing viral replication and host defense optimization, constructing a multi-dimensional molecular network of non-coding RNA synergistic intervention in VMC.

MicroRNAs (miRNAs) are endogenous small RNA molecules widely present in eukaryotic organisms that regulate post-transcriptional gene expression by binding to target mRNAs. In CVB3-induced myocarditis, specific miRNAs modulate both viral replication and host immune responses through targeted gene and pathway interactions. For instance, miR-203, miR-590-5p, and miR-126 enhance viral replication (74–76), whereas miR-221 and miR-222 demonstrate cardioprotective effects (77). miR-22-3p exhibits dual functionality during CVB3 infection: it suppresses viral RNA translation while paradoxically facilitating late-stage viral replication, with its downregulation of protocadherin-1 (PCDH1) further promoting viral propagation (78). The upregulated maternally expressed gene 3 (MEG3) acts as a competing endogenous RNA (ceRNA) for miR-21, thereby enhancing viral replication. The MEG3/miR-223/TRAF6/NF-κB signaling pathway emerges as a promising therapeutic target for VMC (79). Additionally, miR-30d improves cardiac outcomes in ischemic cardiomyopathy by reducing cardiomyocyte apoptosis through targeting MAP4K4 in heart muscle cells and ITGA5 in cardiac fibroblasts (80).

Current GT target distinct pathological mechanisms, ranging from direct-acting antiviral approaches (e.g., RNA helicase inhibition) to host microenvironment modulation through non-coding RNA regulation. The synergistic application of these interventions enables comprehensive disease management targeting both symptomatic relief and pathogenic resolution. Future advancements require overcoming technical challenges such as precision drug delivery and real-time therapeutic modulation. This will facilitate the establishment of multidimensional gene therapy platforms and accelerate translation from preclinical research to clinical applications.

### 3.4 Mesenchymal stem cells therapy

MSCs exhibit multidimensional therapeutic potential in VMC management. In both CVB3-induced and autoimmune myocarditis models, MSCs administered via intramuscular or intravenous routes significantly mitigate disease progression. This therapeutic effect manifests as reduced myocardial tissue damage, improved cardiac systolic function, and decreased inflammatory cell infiltration (81). These therapeutic effects primarily stem from MSC-mediated paracrine immunoregulation, involving two synergistic mechanisms: nitric oxide (NO)-dependent viral replication suppression and interferon-gamma (IFN-γ)-associated preactivation inhibition. This dual action effectively blocks CVB3-induced cardiomyocyte apoptosis, oxidative stress, and viral biosynthesis/release (82). MSCs administration downregulates proinflammatory cytokines (e.g., TNF-α, IL-6) while modulating cardiac monocyte activation patterns enhancing anti-apoptotic signaling pathways (83). MSCs simultaneously target virus-host interactions and fine-tune innate immune responses, thereby demonstrating myocardial protective capabilities. These dual actions establish a scientific foundation for developing precision stem cell-based therapies against viral myocarditis (VMC).

### 3.5 Antioxidant therapy

Oxidative stress (OS) represents a pathophysiological condition resulting from disrupted redox equilibrium between pro-oxidant and antioxidant systems, marked by reactive oxygen species (ROS) overproduction due to impaired peroxide metabolism (84). This redox imbalance initiates pathological cascades involving neutrophilic inflammation, dysregulated protease activity, and oxidative intermediate deposition, all of which exacerbate cellular damage. Study confirms OS serves not only as a fundamental mechanism of biological aging but also constitutes a critical pathological driver across multiple disease states (85). Antioxidants counteracting this process maintains redox homeostasis by scavenging free radicals and neutralizing their cytotoxic effects, thereby playing essential regulatory roles in oxidative balance (86, 87).

In the management of OS associated with VMC, antioxidant therapy plays a critical role, particularly in modulating inflammatory responses and preserving cellular function. Penfluridol (PF) demonstrates both anti-inflammatory and antioxidant properties in lipopolysaccharide (LPS)-activated macrophages. Specifically, PF suppresses NLRP3 inflammasome activation, consequently decreasing secretion of pro-inflammatory cytokines including TNF-α and IL-6 (88). Concurrently, through activation of the Nrf2/HO-1 signaling pathway, PF enhances superoxide dismutase (SOD) activity while reducing malondialdehyde (MDA) levels, effectively alleviating OS (89). These combined mechanisms substantially attenuate LPS-induced macrophage injury.

Beyond revealing PF’s dual-axis modulation of NLRP3/Nrf2 pathways in regulating inflammatory-oxidative equilibrium, the study demonstrated that sulfhydryl-containing antioxidants possessed distinct cardioprotective mechanisms with extended therapeutic promise in VMC. In CVB3-infected murine models, the thiol-based antioxidants captopril and N- (2-mercaptopropionyl)glycine (MPG) significantly improved survival rates, reduced myocardial injury markers (including cellular infiltration, necrosis, and calcification), and suppressed infection-induced upregulation of myocardial manganese superoxide dismutase (Mn-SOD) and copper-zinc superoxide dismutase (Cu/Zn-SOD) mRNA expression (90).

Antioxidants mitigate myocardial injury through scavenging ROS and modulating inflammatory/OS pathways. These findings establish a comprehensive experimental foundation for developing targeted therapeutic strategies against VMC with OS regulation as the core mechanism.

## 4 The treatment of VMC induced by other viruses

### 4.1 Coronavirus induced-myocarditis

The pathological mechanisms underlying COVID-19-associated VMC caused by SARS-CoV-2 remain incompletely understood. Evidence indicates that glucocorticoids combined with IVIG demonstrate clinical utility in such cases. While the use of glucocorticoids in VMC caused by CVB3 remains controversial, they may exert a myocardial-protective effect through combined immunosuppressive, anti-inflammatory, and anti-shock mechanisms (91). IVIG, with its dual antiviral and anti-inflammatory properties, has been shown to significantly improve outcomes in VMC when administered early and in adequate doses. The European Society of Cardiology (ESC) guidelines explicitly recommend evaluating a glucocorticoid-IVIG combination therapy strategy in high-risk patients (92).

A representative case study documented a male patient with SARS-CoV-2-induced VMC complicated by cardiogenic shock and pulmonary infection. Following treatment with methylprednisolone pulse therapy combined with IVIG, the patient demonstrated marked symptomatic improvement along with complete restoration of cardiac architecture and systolic function. Concurrently, myocardial injury biomarkers (troponin I and B-type natriuretic peptide) returned to normal levels (91). This clinical evidence provides compelling support for the critical importance of early combined immunomodulatory therapy in managing coronavirus-related myocarditis.

Given the absence of clinically approved SARS-CoV-2-specific vaccines and targeted antiviral therapies (93), the development of protease inhibitors targeting the viral main protease (Mpro) has emerged as a critical priority in antiviral research. FRET-based screening platforms have identified potent Mpro inhibitors including boceprevir and GC-376, which exhibit significant antiviral activity with IC_50_ values ranging from single-digit to submicromolar concentrations. These compounds demonstrated efficacy in cellular infection models, with EC50 values ranging from 0.49 to 3.37 μM. Structural characterization via X-ray crystallography (2.15 Å resolution) revealed GC-376’s unique R/S dual-conformation binding mode within the protease active site. This finding provides a structural framework for rational lead compound optimization (94). Therapeutically, these protease inhibitors may serve as monotherapies or combine with RNA polymerase inhibitors such as remdesivir, enhancing therapeutic outcomes through multitarget synergistic effects while mitigating resistance development risks.

Emerging research demonstrates that N^5^-methylcytidine (m^5^C) RNA modifications serve as key regulatory mechanisms in SARS-CoV-2 pathogenesis. This evolutionarily conserved epigenetic modification orchestrates multiple pathophysiological processes including embryonic development, tumor progression, and viral infection through modulating RNA stability, nucleocytoplasmic trafficking, and translational efficiency (95, 96). Within COVID-19-induced myocardial injury, m^5^C methylation drives disease progression via a tripartite pathogenic mechanism: (1) controlling viral genomic stability, (2) fine-tuning cytokine storm magnitude, and (3) mediating ACE2/angiotensin axis signaling. Crucially, experimental data confirm that hyperactivated m^5^C methyltransferases induce innate immune dysregulation and potentiate cardiomyocyte damage (97).

Despite notable advancements in current studies, several pivotal scientific challenges remain to be addressed: First, the relationship between the therapeutic time window and dose-response dynamics of glucocorticoid administration. Second, optimizing the pharmacokinetic profiles of Mpro inhibitors for clinical translation. Third, elucidating the precise regulatory mechanisms underlying the m^5^C methylation network in myocardial tissue. The development of multi-omics analytical platforms and organoid-based models will serve as crucial breakthroughs for unraveling SARS-CoV-2-induced myocarditis mechanisms and advancing novel therapeutic approaches.

### 4.2 Adenovirus induced-myocarditis

Adenoviral myocarditis exhibits significant clinical heterogeneity. Early manifestations often mimic upper respiratory or gastrointestinal infections, yet rapid progression to fulminant heart failure remains a critical concern. Current clinical management relies primarily on supportive interventions: strict bed rest (≥3 months acutely), high-dose vitamin C for free radical scavenging, glucocorticoid/immunoglobulin therapy to counter hyperactive immunity (98), and mechanical circulatory support (e.g., ECMO) in severe cases. Antiviral agents such as ribavirin and interferon demonstrate limited efficacy due to narrow therapeutic windows and target non-specificity. Notably, these therapeutic constraints stem from insufficient mechanistic understanding of early viral cytopathic effects-particularly AdV serotype 5 (Ad5)-mediated disruption of cardiac electrophysiological homeostasis.

Preclinical breakthroughs reveal AdV serotype 5 (Ad5) pathogenesis mechanisms. Mechanistic studies establish that Ad5 suppresses GJA1 transcription via β-catenin-dependent pathways, consequently downregulating connexin 43 (Cx43) protein. The early viral protein E4orf1 induces β-catenin phosphorylation, effectively inhibiting Cx43 gene transcription. During initial Ad5 infection, Cx43 phosphorylation triggers electrophysiological abnormalities. Super-resolution imaging confirms dissociation of the Cx43/ZO-1 complex initiates gap junction remodeling in infected cardiomyocytes (99), ultimately provoking arrhythmias through impaired intercellular communication.

Existing therapies lack precision targeting. Future strategies should integrate multi-omic subtyping to develop combinatorial approaches, coupled with immunophenotype-guided individualized interventions. Key translational bottlenecks involve enhancing cardiac-targeted delivery efficiency and validating long-term safety in large-animal models.

### 4.3 PVB19-induced myocarditis

PVB19-induced myocarditis demonstrates marked clinical heterogeneity. This condition can progress rapidly to fulminant heart failure or cardiogenic shock, particularly in pediatric and adolescent populations where case fatality reaches 50-80%. Clinical management centers on supportive interventions: strict bed rest, mechanical circulatory support (e.g., ECMO/IABP), and respiratory assistance. Some clinical studies have shown complete clinical recovery after ECMO support combined with intravenous IVIG, but the efficacy of intravenous IVIG for patients with high viral load is still controversial (100).

Future strategies require multi-omic subtyping integration to develop individualized combination approaches. Concurrent optimization of IVIG timing and dosing regimens is essential. Critical translational barriers include enhancing cardiac-targeted delivery efficiency and validating long-term therapeutic outcomes in large-animal models.

## 5 Future and prospect

Although significant advances have been made in recent years in understanding the pathophysiology and treatment of myocarditis, further research remains critical. Treatment of myocarditis requires a thorough evaluation of the patient’s etiology, underlying pathophysiological mechanisms, and clinical manifestations.

Future research should prioritize the following strategic directions: (1) conduct comprehensive investigations into dynamic immune cell subset interactions (particularly CD8^+^ T lymphocytes and neutrophils) across disease progression phases; (2) design spatiotemporal-specific modulation strategies leveraging non-coding RNAs (microRNAs, lncRNAs); (3) enhance clinical translation of pathogen-specific vaccines and broad-spectrum antivirals including Mpro inhibitors; (4) innovate multimodal combination therapies (e.g., CRISPR-based editing coupled with stem cell engineering) to concurrently suppress viral proliferation and immune dysregulation; (5) implement multi-omics integration (single-cell transcriptomics, metabolic profiling) with organoid platforms to decode virus-host interface networks and enable precision subclassification with personalized regimens. Concurrently, longitudinal outcome optimization requires focused management of chronic inflammatory cascades and fibrotic transitions, aiming to transcend symptomatic palliation toward definitive disease modification.
